# Assessment of the vegetation sensitivity index in alpine meadows with a high coverage and toxic weed invasion under grazing disturbance

**DOI:** 10.3389/fpls.2022.1068941

**Published:** 2022-11-23

**Authors:** Yi Hu, Xiaowei Gou, Atsushi Tsunekawa, Yunxiang Cheng, Fujiang Hou

**Affiliations:** ^1^ School of Ecology and Environment, Inner Mongolia University, Hohhot, China; ^2^ Key Laboratory of Ecology and Resource Use of the Mongolian Plateau, Ministry of Education of China, Hohhot, China; ^3^ Department of Grassland Resource and Ecology, College of Grassland Science and Technology, China Agricultural University, Beijing, China; ^4^ Arid Land Research Center, Tottori University, Hamasaka, Tottori, Japan; ^5^ College of Pastoral Agriculture Science and Technology, Lanzhou University, Lanzhou, China

**Keywords:** vegetation health assessment, grazing, alpine meadows, vegetation index, VOR, palatable species

## Abstract

Maintaining healthy ecosystems is essential to ensure sustainable socio-economic development. Studies combining remote sensing data with grassland health assessments, extensively performed at different scales, are important for monitoring grassland health from a spatiotemporal perspective to enable scientific grazing management. However, most studies only use quantitative grassland degradation indices, such as grassland cover; this is done despite the fact that some degraded grasslands maintain a high level of cover solely by virtue of the proliferation of toxic weeds. Thus, seeking indices that are a more accurate representation of the health status of grassland vegetation is of utmost importance. Therefore, in order to accurately characterize the ecological integrity of grasslands (i.e., while limiting the impact of confounding variables such as weeds), we chose the grassland health comprehensive evaluation index VOR (vigor, organization, and resilience) to assess the health of grasslands on the Tibetan Plateau. We applied the VOR evaluation indices to two rangelands with different grazing intensity on the Tibetan Plateau, and extracted 11 commonly used vegetation indices based on remote sensing images of rangelands,then modeled them with the data from field surveys. Our results show that the FVC, PS, and VOR were higher in lightly grazed pastures than in heavily grazed pastures in the 2017 and 2018 growing seasons. At the beginning of the sampling period, Poaceae accounted for a greater proportion in the HG pasture. However, by August 2018, the proportion of Poaceae in the LG pasture exceeded that in the HG pasture. the proportion of Forbs in the HG pasture was significantly greater than that in the LG pasture. This indicates that vegetation response to grazing disturbance is not only a volume reduction but also a vegetation composition change. The ratio vegetation index was the most sensitive to the vegetation health response, enabling the quantification and prediction of regional vegetation health and objectively reflecting the actual condition of the grassland ecosystem. According to a multiple regression analysis, the main climatic limiting factor in the region is precipitation, which positively correlated with VOR; whereas, grazing disturbance is an important driving factor, and it is inversely correlated with VOR.

## 1 Introduction

Grazing can directly alter the structure and composition of plant communities, resulting in reduced aboveground biomass, a reduction in the species richness of palatable grass species, and increase in the species richness of unpalatable grasses (Provenza et al., 2003; Wu et al., 2009; Dai et al., 2019; Dai et al., 2021). The dictionary definition of palatable is ‘pleasant to the taste’, and in this sense, describes an inherent characteristic of a plant (Hodgson, 1979). Selective grazing of preferred plants and their resulting inability to out-compete less palatable species is assumed to be the main factor driving range deterioration and limiting range improvement (Ellison, 1960). Alpine meadows, a major vegetation type widely distributed across the central-eastern Tibetan Plateau (TP), play a variety of roles in maintaining ecosystems (Pepin et al., 2015; Yang et al., 2017; Wang et al., 2021). The TP alpine meadows have unique ecological features, such as a simple structure with a poor restoration capacity and stability when exposed to external disturbances (Wang et al., 2019). Additionally, these meadows are susceptible to the negative effects of overgrazing (Dai et al., 2019). In overgrazed grasslands, unpalatable plant species (mainly poisonous forbs) often grow vigorously and expand their ranges, and this is a condition indicative of degradation succession (Tuvshintogtokh & Ariungerel, 2013). This outcome of the degradation succession is a degraded grassland that may still have a high vegetation cover and may be falsely classified as “healthy” if judged by cover alone. Therefore, it is necessary to analyze grassland degradation from two perspectives: quantity and quality of forage (Lyu et al., 2020).

Remote sensing provides spectral, temporal, and spatial data that can be acquired from large areas simultaneously, keeping field-laboratory efforts to a minimum, ultimately facilitating efficient grassland management and conservation (Guerini Filho et al., 2019). Most currently methods are linear models based on the normalized difference of vegetation index (NDVI); these models are fueled by indicators such as the net primary productivity, FVC, and above-ground biomass (AGB) to investigate the driving forces affecting grassland degradation (Sun et al., 2017; Zhang et al., 2019). This biomass-based monitoring approach fails to precisely reflect the complexity underlying the grassland degradation process and its diverse manifestations. Few studies have considered the selective foraging by livestock to qualitatively evaluate the health of grasslands. Accordingly, exploring comprehensive indicators that reflect the health of grassland vegetation is urgent. Costanza’s (Costanza & Mageau, 1999) three ecosystem indicators— vigor, organization, and resilience (VOR)— constitute a widely accepted ecosystem health index (Ainslie, 1994). The VOR health assessment index play an important role in providing qualitative and quantitative data about ecosystem attributes and rangeland management. Li et al. (Li et al., 2013) calculated the health indices of four grassland plots at different levels of degradation in alpine meadows on the Tibetan Plateau by measuring their VOR, and the results indicated that the VOR decreased in a consistent manner across the four plots along the disturbance gradients. The VOR health assessment index play an important role in providing qualitative and quantitative data about ecosystem attributes and rangeland management. Li et al. (Li et al., 2013) calculated the health indices of four grassland plots at different levels of degradation in alpine meadows on the Tibetan Plateau by measuring their VOR, and the results indicated that the VOR decreased in a consistent manner across the four plots along the disturbance gradients. The health of different types of grassland ecosystems has been evaluated at different scales (Wang et al., 2010; Li et al., 2013; Shen et al., 2016; Yan et al., 2016; Peng et al., 2017). Despite difficulties underlying the process of acquiring direct measurements, efforts have been made to quantify ecosystem health. The VOR indices allow not only the quantitative monitoring of grassland vegetation but also the qualitative assessment of vegetation health, thus providing a good indication of the impact of climatic factors and grazing disturbances on vegetation. Remote sensing monitoring of grassland degradation is mainly done by establishing a relationship between the vegetation index (VI) and grassland degradation evaluation indicators. The VI is a mathematical combination of different bands of an electromagnetic spectrum; these bands are indicative of photosynthetic activity and vegetation vigor, and these variables are used as proxies for vegetation time-series analysis, condition monitoring, and change (Brantley et al., 2011; Hill et al., 2013). Various vegetation indices (VIs), especially the NDVI, have been used to model grassland biomass. Other VIs, such as the modified soil-adjusted vegetation index (MSAVI) and the enhanced vegetation index (EVI), have also been developed for characterizing different vegetation-linked dynamics. For degraded grasslands with high vegetation cover (i.e., in Tibetan Plateau alpine meadows), there is no consensus on which commonly used VIs are better predictors of their health changes and spatial layout. Therefore, it is necessary to select sensitive remotely sensed vegetation indices to accurately assess the vegetation health of the alpine meadows while circumventing the confounding effects of invasive toxic weeds.

## 2 Materials and methods

### 2.1 Study area

This study was conducted in Dangluo Township (99°09′–100°40’ E, 33°43’–34°37’ N), a Township on the Tibetan Plateau (TP), in the northeastern part of Guoluo Tibetan Autonomous Prefecture, Qinghai Province. The average altitude of this area is between 2900 m and 4500 m, and the area is also characterized by a cold and humid continental climate, with an average annual temperature below -4°C, an annual precipitation of 565.9 mm, and average yearly sunshine exceeding 2,500 h; additionally, it has no absolute frost-free periods. A 16.5 km2 area around the settlement of Dangluo Township was delineated for this study. Dangluo Township is in the upper reaches of the Yellow River on the TP, in a mountainous valley, and is part of the “Three Rivers Source” ecological reserve. The region is dominated by alpine meadows, with dominant species such as Kobresia humilis, Stipa sp., and Carex sp., and associated species such as Potentilla chinensis, Leontopodium nanum, Thalictrum alpinum, Polygonum viviparum, and Pedicularis chinensis.

**Figure 1:**
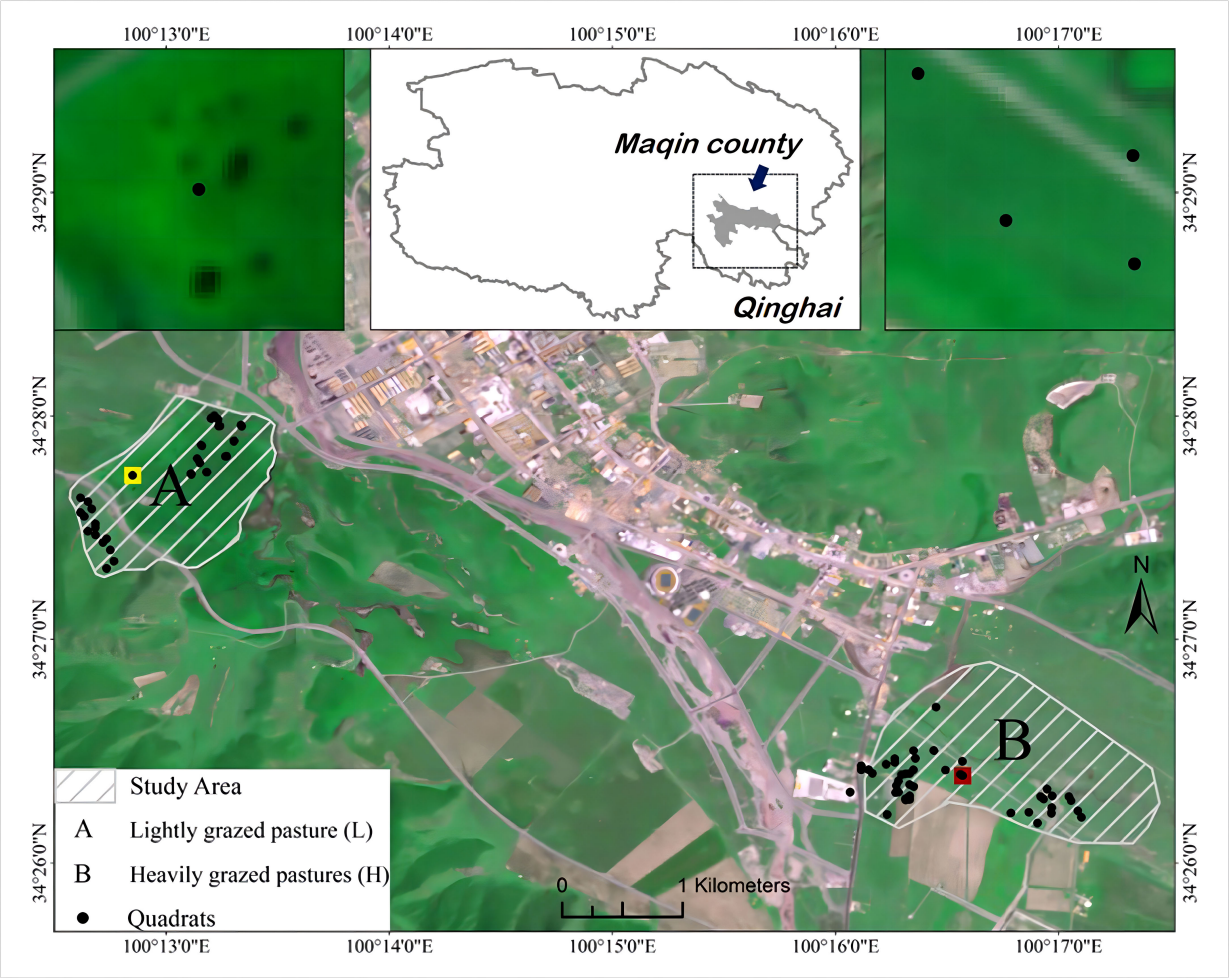
Design of the sample plots.

Two alpine meadow pastures, mainly grazed by yaks, were selected as sample sites (**Figure 1**). Two pastures were selected, and they were similar in: type (alpine meadows), production and utilization patterns (yak grazing), and the growth level of grasslands. That is, the yield of edible forage is theoretically equal on the grassland and has the same growth cycle, with no other disturbances other than grazing. Based on the number of grazing yaks and the area of grassland grazing in the pasture, the grazing intensity of the two pastures was calculated, and then the relative grazing intensity of the two pastures of the pasture was calculated using the following formula: Relative grazing intensity = total number of yaks in pastures/area of different grazing gradients. The number of livestock and the grassland area of the two pastures were investigated, and those whose grazing intensity (GI) was 1.68 and 2.17 heads/hm2 were classified as light and heavy grazing (LG and HG), respectively.

### 2.2 Data source and processing

Vegetation surveys were conducted in two pastures in 2017 and 2018, and 9–16 samples with an area of 1 m × 1 m were collected in July and August. Samples were collected by cutting the above-ground parts of the plant flush using the mowing method and recording community information, such as the name, cover, and height of the main species in the sample square. Those parts were weighed fresh and then baked at 70°C to a constant weight and then weighed dry. Finally, 34 and 42 samples were collected from LG and HG pastures, respectively. The importance values for each species within each pasture quadrat were calculated, and their Shannon-Wiener Index (H) was determined using the following equation:


(1) $$Pi=(R_{h}+R_{c}+R_{f})/3$$

(2) $$H=-\sum_{i=1}^{S}(Pi\ln Pi)$$

A distinction was made between palatable and unpalatable species, and the proportion of palatable species (PS) was calculated using the following equation:


(3) $$PS=\sum Pi_{PS}\ln Pi_{PS}/\sum Pi\ln Pi$$

Where *Pi, R_h_, R_c_, R_f_, H, S* and *Pi_ps_* represent the importance values, relative height, cover, frequency, Shannon-Wiener diversity index, the total number of species within each quadrat, and the importance value of each PS, respectively. Where *R_h_ = H_i_/H, R_c_ = C_i_/C, R_f_= F_i_/F, 0< H<*1n *S.*

For the remote sensing data, the following four phases of data were acquired to match the ground survey data in time: Gaofen-2 satellite images with a spatial resolution of 0.8 m collected in July 2017; SPOT6 satellite images with a spatial resolution of 1.5 m collected in August 2017; ZY-2 satellite images acquired in July 2018 with a spatial resolution of 2.36 m; ZY-3 satellite images acquired in August 2018 with a spatial resolution of 2.1 m. The above remote sensing images were processed using ENVI 5.3 (Exelis Visual Information Solutions, USA). The pre-processing, such as radiometric calibration and atmospheric correction, were conducted first, and then 11 VIs were calculated using the band math tool. The formulas are shown in **Table 1**. ArcGIS 10.7 (Environmental Systems Research Institute, CA, USA) was used to extract the VIs of the corresponding location raster based on the GPS position coordinates recorded at each sample site. Subsequently, NDVI was used to calculate the FVC using equation 4:


(4) $$FVC=(NDVI-NDVI_{soil})/(NDVI_{veg}-NDVI_{soil})$$

**Table 1:** Vegetation index formula.

Vegetation index	Designation	Formula
NDVI	Normalized difference vegetation index	$\frac{\rho_{NIR}-\rho_{RED}}{\rho_{NIR}+\rho_{RED}}$
EVI	Enhanced vegetation index	$2.5(\frac{\rho_{NIR}-\rho_{RED}}{\rho_{NIR}+6\rho_{RED}-7.5\rho_{BLUE}+1})$
RVI	Ratio vegetation index	$\frac{\rho_{NIR}}{\rho_{RED}}$
TVI	Transformed vegetation index	$\sqrt{NDVI+0.5}$
GNDVI	Green normalized difference vegetation index	$\frac{\rho_{NIR}-\rho_{GREEN}}{\rho_{NIR}+\rho_{GREEN}}$
RDVI	Renormalized difference vegetation index	$\sqrt{NDVI\times RVI}$
SAVI	Soil-adjusted vegetation index	$\frac{1.5(\rho_{NIR}-\rho_{RED})}{\left(\rho_{NIR}+\rho_{RED}+0.5\right)}$
MSAVI	Modified soil-adjusted vegetation index	$\frac{2\rho_{NIR}+1-\sqrt{2{\left(\rho_{NIR}+1\right)}^{2}-8(\rho_{NIR}-\rho_{RED})}}{2}$
OSAVI	Optimized soil-adjusted vegetation index	$\frac{\rho_{NIR}-\rho_{RED}}{\rho_{NIR}+\rho_{RED}+0.16}$
ARVI	Atmospherically resistant vegetation index	$\frac{\rho_{NIR}-\rho_{RED}+\theta (\rho_{BLUE}-\rho_{RED})}{\rho_{NIR}+\rho_{RED}-\theta (\rho_{BLUE}-\rho_{RED})}$
IPVI	Infrared percentage vegetation index	$\frac{\rho_{NIR}}{\rho_{NIR}+\rho_{RED}}$

The ***NDVI*** values with a cumulative probability of 5% and 90% were considered ***NDVI_soil_*** and ***NDVI_veg_***, respectively.

Outliers are removed based on a normality assessment of the data using SPSS 23. The differences in forage quality of the two pastures in different years and the same period but different grazing intensities were compared by one-way analysis of variance (ANOVA). was performed after the normality tests. Correlation analysis was performed in R Studio 4.2.0 for each grassland assessment indicator of vegetation health and 11 vegetation indices. Subsequently, cumulative temperature versus average precipitation is calculated using the temperature and precipitation of the first 12 months of the month in the field survey. Multiple linear regression and curve estimation were performed using vegetation index (based on remote sensing imagery) as independent variables, and VOR, AGB, FVS, PS as dependent variables. Statistical model assessment and comparison was performed in R Studio 4.2.0 by comparing the six statistical parameters AIC, BIC, R^2^_adjusted, RMSE and Sigma. Finally, the selected sensitivity indices were submitted into the linear regression model established using the measured values of VOR. The VOR of the whole study area was analyzed, being divided into four classes. The area of each class was determined using ENVI 5.3.

### 2.3 Construction of the evaluation system

#### 2.3.1 Reference system

Identifying the reference system is the first step in grassland health evaluation. In ecosystem health evaluation, a completely healthy ecosystem is usually selected as a control (Hou et al., 2004). Here, a 4-year enclosure area near the study site, which was undisturbed by grazing and, thus, less damaged, was selected as the reference system.

#### 2.3.2 Indicator selection and calculation

Both qualitative and quantitative assessments are important when selecting indicators for assessing grassland health (Önal et al., 1998). Vigor is a measure of its activity, metabolism, or primary productivity (Costanza, 2012). The formula used is as follows:


(5) $$V=V_{x}/V_{ck}$$

where *V_x_* and *V_ck_* are the AGB of the evaluated and the reference plots, respectively.

Organization refers to the interactions occurring between the components of a system (Costanza, 2012). Here, the community species diversity (H) was considered a community organization parameter, using equation 6:


(6) $$O=O_{x}/O_{ck}$$

where *O_x_* and *O_ck_* are the H of the evaluated and the reference plots, respectively.

Resilience is the ability of an ecosystem to withstand destructive pressures. Grassland health restoration includes the restoration of productivity and structure (Shan et al., 2012). In rangelands, the main disturbance affecting plant growth is grazing (Li et al., 2013); thus, we chose the proportion of palatable species as an indicator of rangeland quality using equation 7:


(7) $$R=R_{x}/R_{ck}$$

where *R_x_* and *R_ck_* are the PS of the evaluated and reference plots, respectively.

The VOR calculation model is as follows:


(8) VOR=WV×V+WO×O+WR×R

where *WV + WO + WR =* 1, *WV, WO, WR* ≥ 0. Where *WV*, *WO*, and *WR* are the weight coefficients of *V*, *O*, and *R* respectively. *VOR*∈[0, 1].

#### 2.3.3 Weight indicator

A good weight measure should be comprehensive and a subjective and objective reflection of the relative importance of indicators in decision-making or evaluation (Ye et al., 2011). Here, the entropy method was used to determine the weight of the above indexes. The method relies on probabilistic and statistical analyses to describe the degree of disorder in the system (Wu et al., 2017).

The steps of the entropy method are as follows:

1) Standardizing the raw data.

Assuming m and n represent the number of indicators and samples, respectively, we determined the value of the j index corresponding to the i sample (i = 1, 2, …, n; j = 1, 2, …, m). The original matrix is denoted as R = ( *r*_*ij*_ )m×n. The standardized matrix is S = ( *s*_*ij*_ )m×n; S∈ [0,1]. We converted the absolute value to a relative one: *X*_*ij*_ = | *X*_*ij*_ | using equation 9:


(9) $$S_{ij}=\frac{r_{ij}{-}_{j}^{\min}\{r_{ij}\}}{{\;}_{j}^{\max}\{r_{ij}\}{-}_{j}^{\min}\{r_{ij}\}}$$

2) Entropy determination:


(10) $$e_{j}=-K\sum_{i=1}^{n}P_{ij}\ln P_{ij}$$

Where $P_{ij}=\frac{sij}{\sum_{i=1}^{n}sij}$ and *K*=1/*ln*(*n*) 3) Determination of weights:


(11) $$w_{j}=\frac{d_{j}}{\sum_{j=1}^{m}d_{j}}$$

Where *dj*=1−*ej* .

#### 2.3.4 Rating classification

We set thresholds to classify the grassland health status classes after calculating the health evaluation index of grassland ecosystems. Then, we could comprehensively evaluate the grassland health conditions. Here, the health status of grassland ecosystems was classified into four classes using the quadrat method. The relative evaluation criteria are rated as follows: 0< VOR<0.25, crash; 0.25< VOR<0.50, alarm; 0.50< VOR< 0.75, unhealthy; 0.75< VOR<1.00, healthy.

## 3 Results

### 3.1 Variation in vegetation

**Figure 2:**
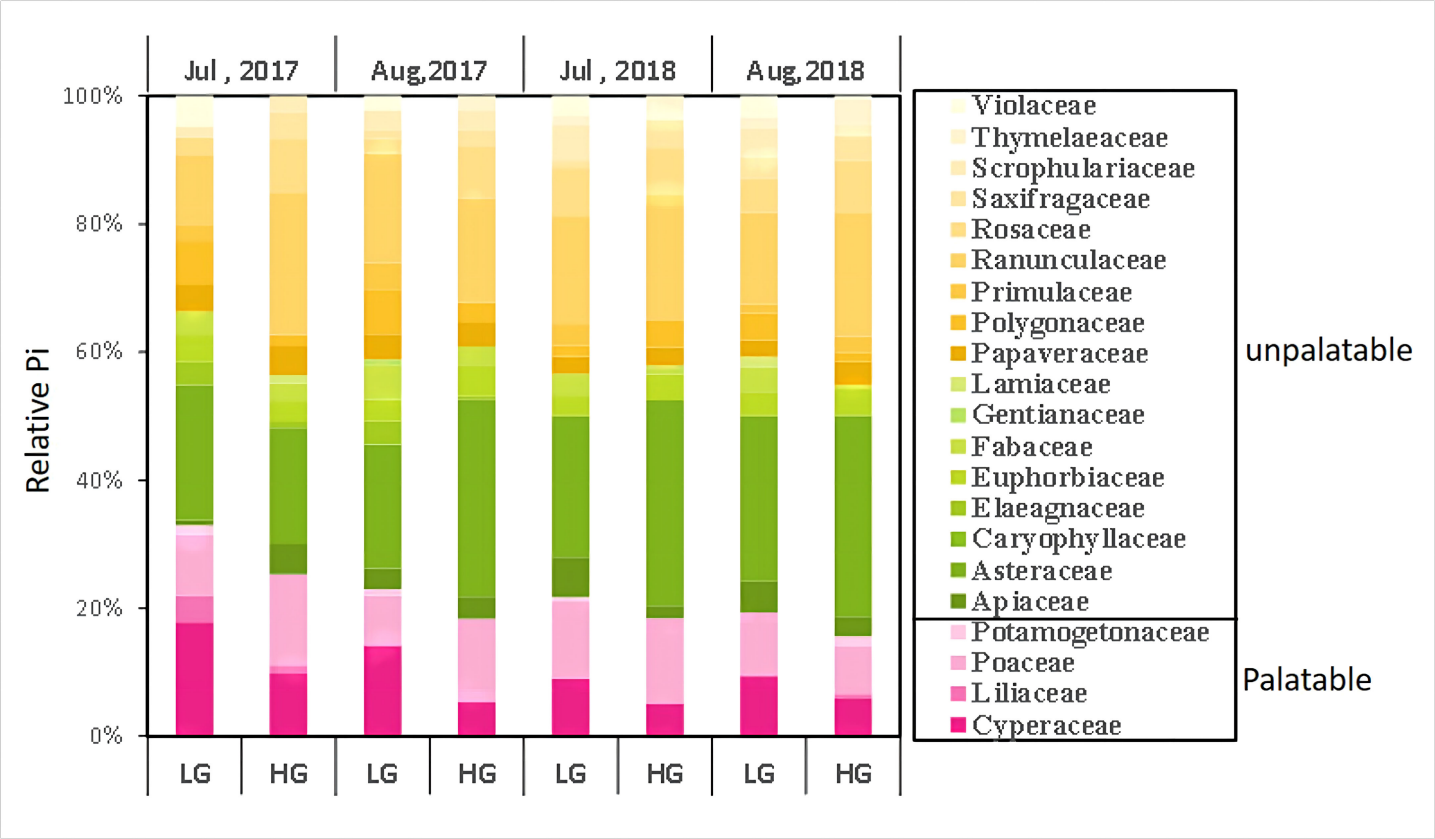
Changes in the proportion of different families in the community.

**Figure 3:**
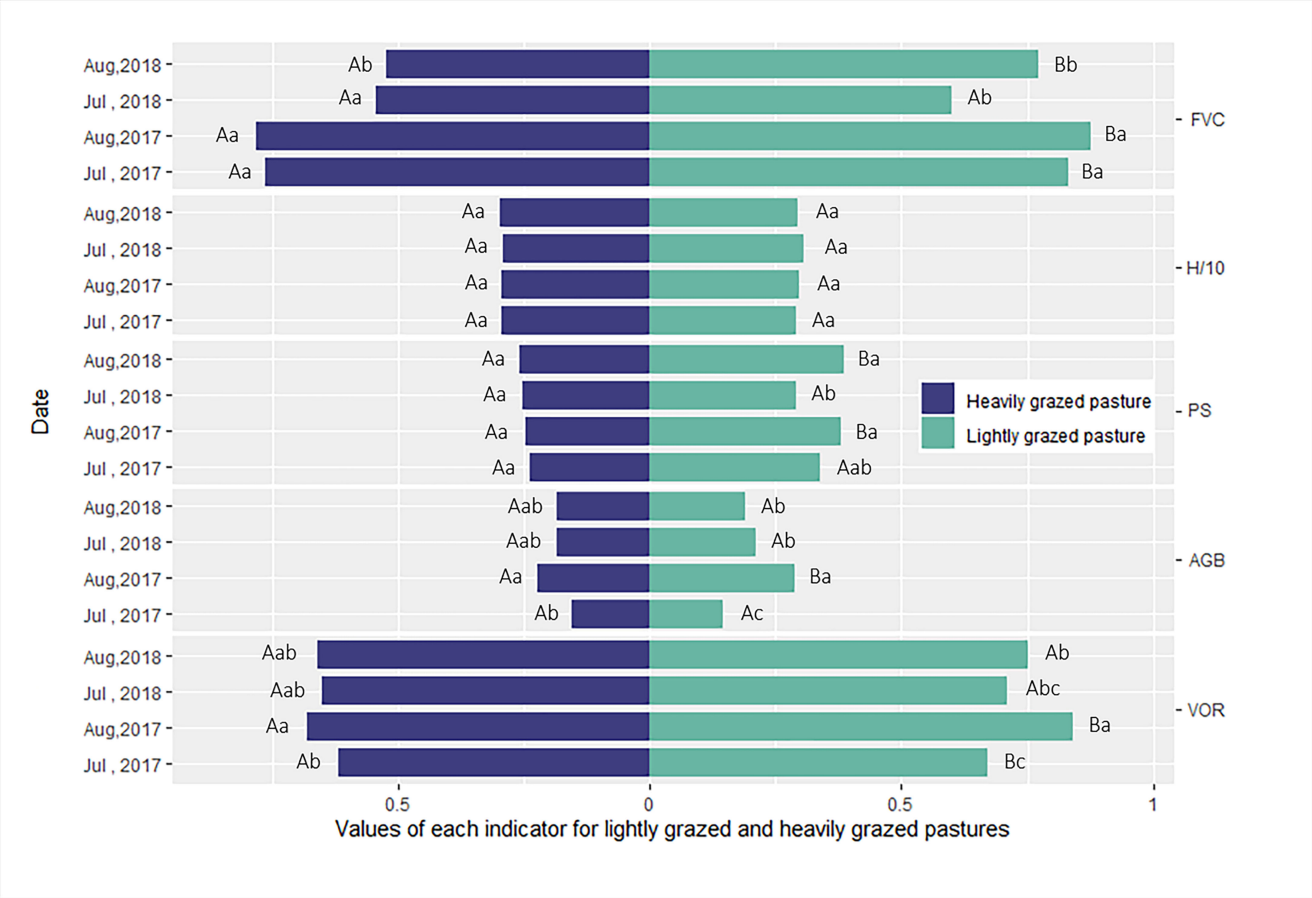
Variation in vegetation cover (FVC), species diversity (H), the proportion of palatable species (PS), above-ground biomass (AGB)(kg/m2), and the composite assessment index of grassland health (VOR) between July and August 2017, and July and August 2018, for lightly (L) and heavily (H) grazed pastures. Different lower-case letters (a, b, and c) represent significant differences in time, and different upper-case letters (A and B) represent significant differences between lightly and heavily grazed pastures.

The proportion of different families within the grassland communities of the two pastures at different times showed that sedge such as *Carex* sp., *Kobresia* sp. and *Scirpus* sp., which are preferred by livestock, were consistently less in HG pastures than in LG. The proportion of sedge in both pastures decreased with time. At the beginning of the sampling period, Poaceae such as *Poa* sp., *Festuca* sp., and *Elymus* sp. accounted for a greater proportion in the HG pasture. However, by August 2018, the proportion of Poaceae in the LG pasture exceeded that in the HG pasture. the proportion of Forbs in the HG pasture was significantly greater than that in the LG pasture, with the Asteraceae and Ranunculaceae being particularly significant (**Figure 2**). As shown in **Figure 3**, the FVC, PS, and VOR were higher in lightly grazed pastures than in heavily grazed pastures in the 2017 and 2018 growing seasons, revealing that grazing adversely affected the health of grassland vegetation in the rangelands of the region. However, there was no significant difference in AGB and diversity between the two pastures. From July 2017 to August 2018, the proportion of palatability species in HG pastures gradually increased from 0.24 to 0.26, and the proportion of palatability species in LG pastures increased significantly from the initial 0.24 to 0.38. The FVC of both pastures decreased significantly with time (*P*< 0.05), AGB increased significantly (*P*< 0.05) in the lightly grazed pastures and non-significantly in the heavily grazed pastures, showing the same trend as VOR (*P<* 0.05). The proportion of palatable forages also increased, implying that the overall health gradually improved, although the rangeland vegetation cover decreased.

### 3.2 Sensitivity index of vegetation health status

**Figure 4:**
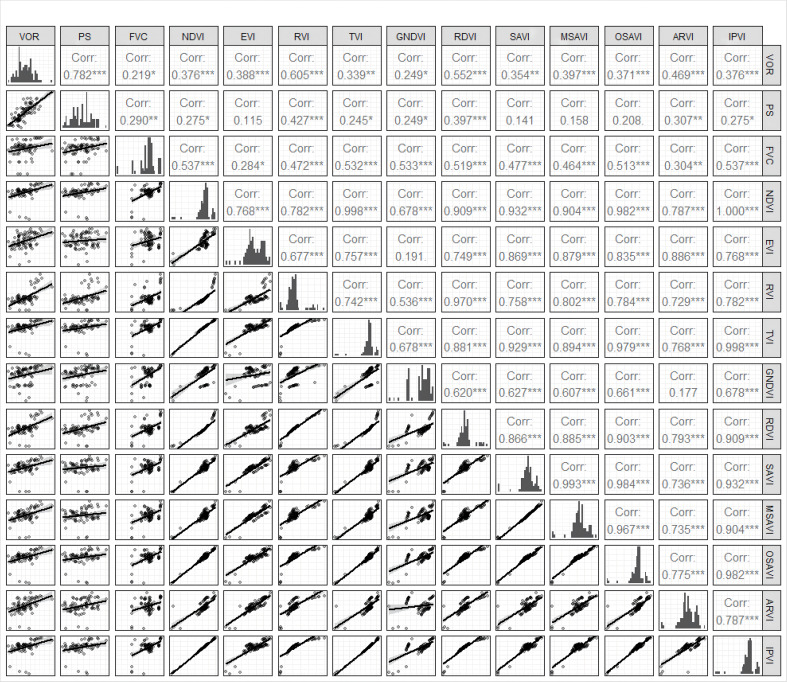
Correlation between indicators and vegetation indices. ***, **, * indicate a significant level of 1%, 5% and 10%, respectively.

**Figure 5:**
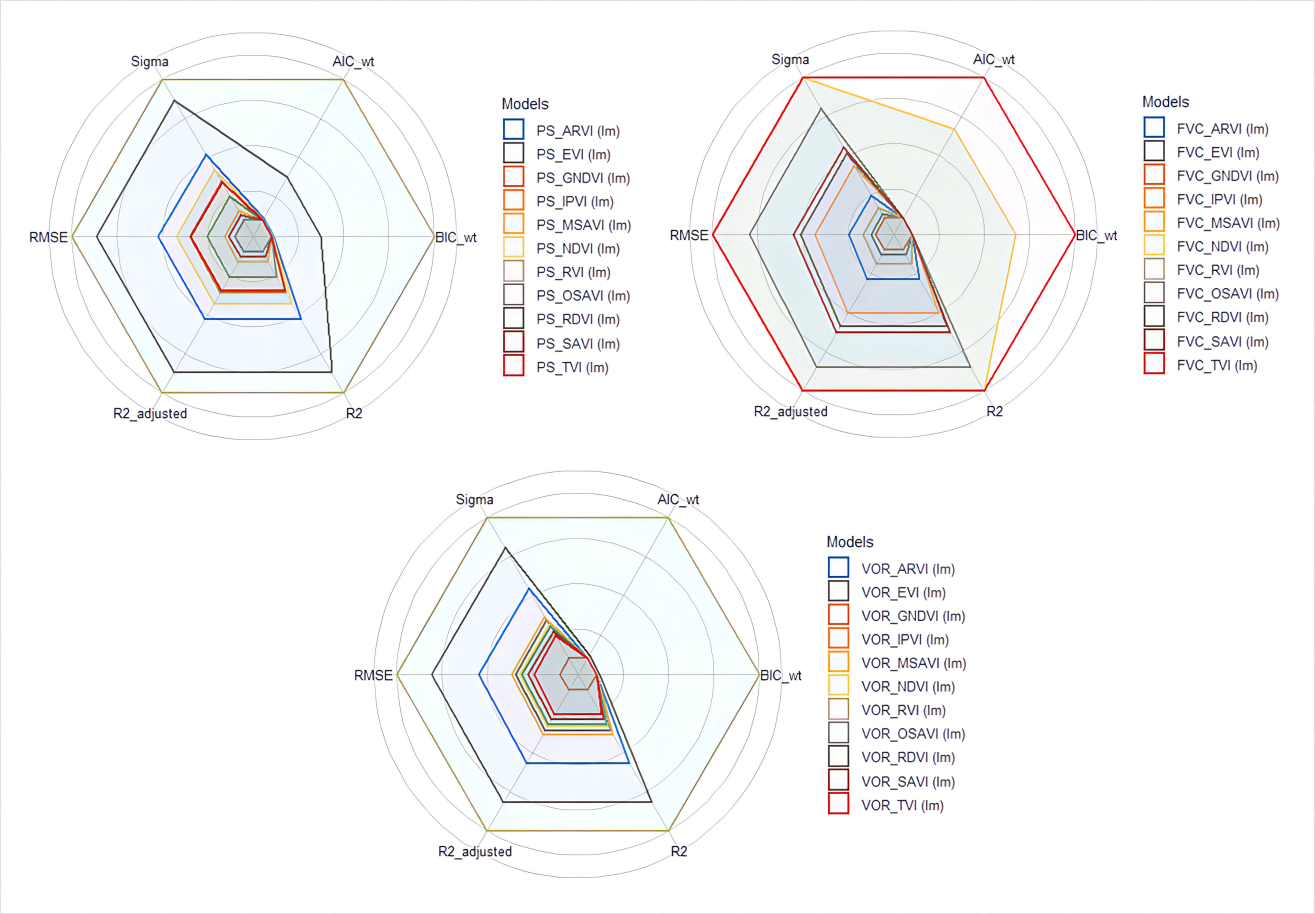
Assessment and comparison of statistical models.

**Figure 6:**
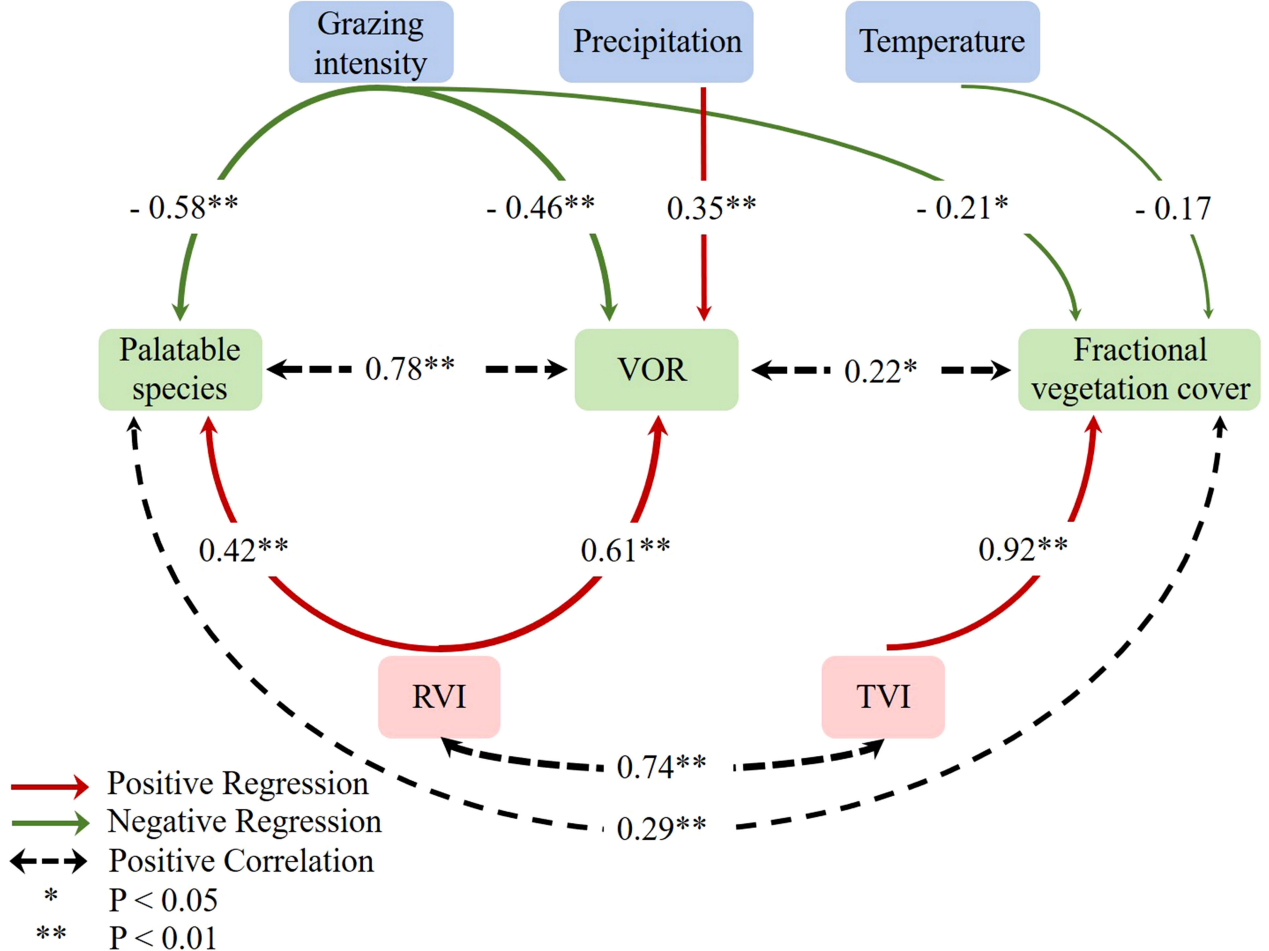
Path analysis shows the effect of climatic factors on vegetation. The width of the arrow is proportional to the strength of the path coefficient.

The PS, FVC, and vegetation health index VOR, which revealed a significant relationship with grazing disturbance in this study, were selected for correlation analysis with respect to 11 vegetation indices combined by remote sensing influence bands. The results showed that two of the eleven vegetation indices correlated with PS (*P*< 0.01), namely RVI (0.427) and RDVI (0.397) (**Figure 4**). EVI and three soil-adjusted vegetation indices were not significantly correlated with PS. All vegetation indices significantly correlated with FVC with the highest correlation between NDVI and IPVI (0.537) (*P*< 0.01). Except for TVI, GNDVI and SAVI that significantly correlated with VOR, all other vegetation indices showed significant high correlations with VOR, with the highest correlation between RVI (0.605) and RDVI (0.552) (*P*< 0.01) (**Figure 4**). These results indicate that most vegetation indices have the potential to reflect FVC and vegetation health index VOR. From the comparison results of the prediction models, RVI was the best prediction index for PS and VOR, and its estimated model for PS had an adjusted R^2^ of 0.172, which was the largest adjusted R^2^ value among all PS estimated models. And the AIC, BIC, RMSE and Sigma value are smaller in this model. This means that the model fits well as the accuracy. In the estimation model of RVI to VOR, the adjusted R^2^ reached 0.357, which was the maximum among all the models, and the AIC, BIC, RMSE and Sigma have the smaller index. Among all the FVC estimation models, TVI was the best because the adjusted R^2^ was the largest of all models at 0.848, while AIC, BIC, RMSE and Sigma are all smaller in this model. (**Table 2**, **Figure 4**). RVI is a sensitive indicator parameter for green plants and correlates well with LAI, dry leaf biomass (DM), and chlorophyll content, and therefore identifies the proportion of community forage well. RDVI, which combined NDVI and RVI, was not as effective as RVI alone in this study. Regression models established for RVI with the proportion of palatable forage in the community and VOR were well fitted, and linear regression models established for TVI with FVC yielded a good fit (**Figure 4**, **Figure 5**, **Figure 6**). Therefore, we chose RVI and TVI to predict VOR, PS, and FVC.

**Table 2:** Model Parameters.

Indicators	Vegetation Index	Function	AIC	AIC weights	BIC	BIC weights	R^2^	R^2^ (adj.)	RMSE	Sigma
PS	NDVI	Linear model	-165.954	0.005	-158.771	0.005	0.076	0.064	0.084	0.085
	EVI	Linear model	-160.661	< 0.001	-153.478	< 0.001	0.013	6.712e-04	0.086	0.088
	RVI	Linear model	-175.851	0.742	-168.667	0.742	0.182	0.172	0.079	0.080
	TVI	Linear model	-164.612	0.003	-157.428	0.003	0.060	0.048	0.084	0.085
	GNDVI	Linear model	-164.777	0.003	-157.594	0.003	0.062	0.050	0.084	0.085
	RDVI	Linear model	-173.485	0.227	-166.302	0.227	0.158	0.147	0.080	0.081
	SAVI	Linear model	-161.211	< 0.001	-154.028	< 0.001	0.020	0.007	0.086	0.087
	MSAVI	Linear model	-161.632	< 0.001	-154.449	< 0.001	0.025	0.013	0.086	0.087
	OSAVI	Linear model	-163.176	0.001	-155.993	0.001	0.043	0.031	0.085	0.086
	ARVI	Linear model	-167.615	0.012	-160.431	0.012	0.094	0.083	0.083	0.084
	IPVI	Linear model	-165.954	0.005	-158.771	0.005	0.076	0.064	0.084	0.085
FVC	NDVI	Linear model	-167.600	0.280	-160.417	0.280	0.848	0.846	0.083	0.084
	EVI	Linear model	-58.023	< 0.001	-50.839	< 0.001	0.413	0.406	0.163	0.165
	RVI	Linear model	-62.086	< 0.001	-54.903	< 0.001	0.442	0.435	0.159	0.161
	TVI	Linear model	-168.504	0.440	-161.320	0.440	0.850	0.848	0.082	0.083
	GNDVI	Linear model	-55.667	< 0.001	-48.484	< 0.001	0.396	0.388	0.165	0.167
	RDVI	Linear model	-98.124	< 0.001	-90.941	< 0.001	0.642	0.638	0.127	0.129
	SAVI	Linear model	-102.930	< 0.001	-95.747	< 0.001	0.663	0.659	0.124	0.125
	MSAVI	Linear model	-89.020	< 0.001	-81.837	< 0.001	0.600	0.595	0.135	0.136
	OSAVI	Linear model	-135.698	< 0.001	-128.515	< 0.001	0.775	0.772	0.101	0.102
	ARVI	Linear model	-69.691	< 0.001	-62.507	< 0.001	0.492	0.486	0.152	0.154
	IPVI	Linear model	-167.600	0.280	-160.417	0.280	0.848	0.846	0.083	0.084
VOR	NDVI	Linear model	-169.567	< 0.001	-162.383	< 0.001	0.141	0.130	0.082	0.083
	EVI	Linear model	-170.424	< 0.001	-163.241	< 0.001	0.150	0.139	0.081	0.082
	RVI	Linear model	-194.085	0.975	-186.901	0.975	0.365	0.357	0.070	0.071
	TVI	Linear model	-167.123	< 0.001	-159.940	< 0.001	0.115	0.104	0.083	0.084
	GNDVI	Linear model	-162.426	< 0.001	-155.242	< 0.001	0.062	0.050	0.086	0.087
	RDVI	Linear model	-186.707	0.024	-179.524	0.024	0.305	0.296	0.074	0.075
	SAVI	Linear model	-168.094	< 0.001	-160.911	< 0.001	0.125	0.114	0.083	0.084
	MSAVI	Linear model	-171.159	< 0.001	-163.976	< 0.001	0.158	0.147	0.081	0.082
	OSAVI	Linear model	-169.220	< 0.001	-162.037	< 0.001	0.138	0.127	0.082	0.083
	ARVI	Linear model	-177.356	< 0.001	-170.173	< 0.001	0.220	0.210	0.078	0.079
	IPVI	Linear model	-169.567	< 0.001	-162.383	< 0.001	0.141	0.130	0.082	0.083

### 3.3 Drivers of vegetation health change

Stepwise regression analysis was performed with PS, FVC, and VOR as dependent variables. Prior to the multiple regression, collinearity diagonostics between the environmental factors was performed. The results showed that the variance inflation factor (VIF) of all environmental factors in the three models were less than 10, indicating that the collinearity among environmental factors was very weak. (HELIL et al., 2019). GI, the cumulative temperature in different months before ground sampling, and mean monthly precipitation with vegetation index (which significantly correlated with PS, FVC, VOR) as independent variables. Results showed that GI exerted highly significant adverse effects on PS and VOR (*P*< 0.01) and a significantly negative effect on FVC (*P<* 0.05) (**Figure 3**, **Figure 6**). Monthly mean precipitation in the two months prior to sampling showed a highly significant positive effect on VOR (*P*< 0.01) (**Figure 6**). Based on these results, we conclude that grazing disturbance affected grassland community composition, thus negatively affecting grassland health. Conversely, precipitation positively affected grassland health.

### 3.4 Spatial patterns of ecosystem indicators

**Figure 7:**
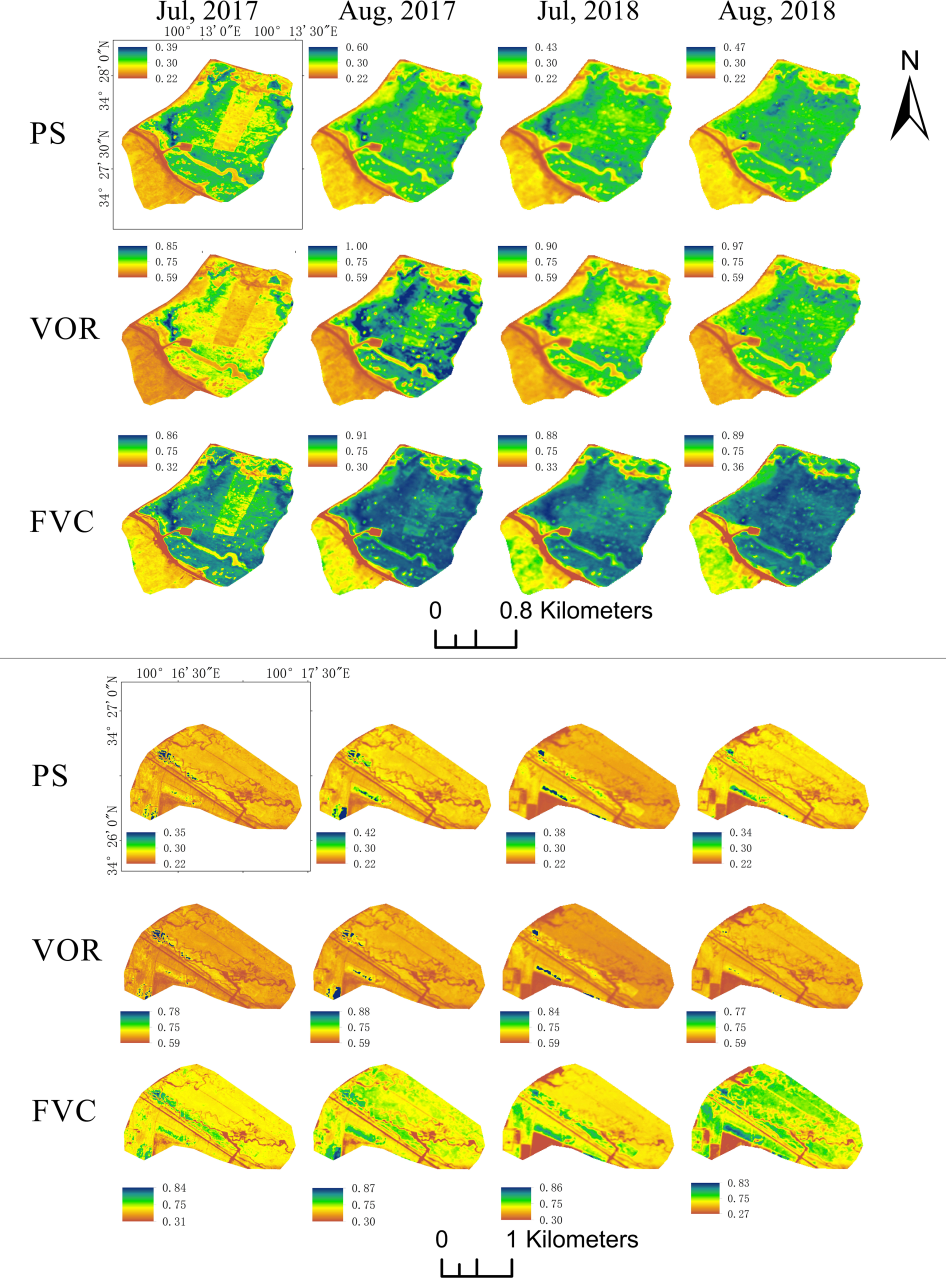
Spatial distribution of the Composite Evaluation Index of Grassland Health (VOR) for light (LG) and heavy (HG) grazing pastures from July to August 2017 and from July to August 2018.

**Figure 8:**
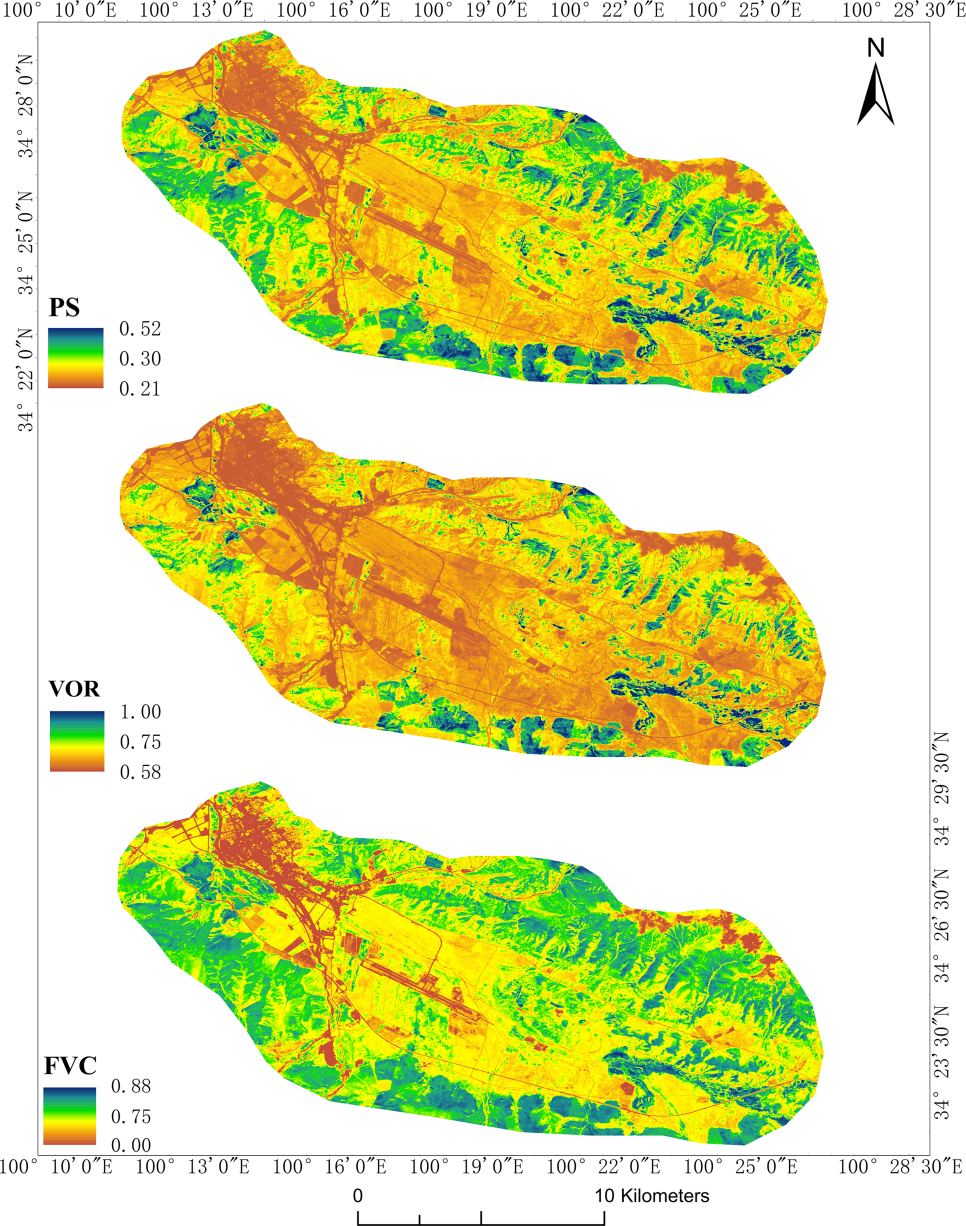
Spatial distribution of the PS, VOR, and FVC in Dangluo Township.

According to the regression analysis results, the overall VOR, PS, and FVC values for lightly grazed pastures were higher than those for heavily grazed pastures. On the spatial scale, the higher VOR was distributed in the north and southeast of the study area for lightly grazed pastures. However, the VOR was lower in the central and southwest regions due to concentrated fenced grazing. PS, FVC, and VOR showed similar spatial distributions. VOR and PS were more evenly distributed spatially in heavily grazed pastures, and the higher FVC was distributed in the southwestern part of the region. Timewise, the proportion of healthy areas with VOR higher than 0.75 in lightly grazed pastures changed from 9% in July 2017 to 60% in August 2018 from 2017 to 2018, with a significant improvement in vegetation health. The same trend was observed for PS and FVC in LG pastures. Vegetation health in the central part of the HG pastures was better than in the surrounding area. Still, the overall health remained poor, with almost always 0% of areas with a VOR exceeding 0.75. However, vegetation health improved slightly from 2017 to 2018 (**Figure 7**). Overall, both pastures had larger areas with FVC values 0.75 greater than VOR, suggesting that despite the correlation between vegetation health and vegetation cover, a high regional FVC does not necessarily imply better vegetation health. Based on the RVI inversion, the VOR of the 16.5 km^2^ area around the settlement of Dangluo Township was obtained, while the FVC was obtained using the TVI. The overall spatial distribution pattern showed that the VOR was lower in the northwest, northeast edge, and central areas (**Figure 8**). The whole region was divided into two classes, namely healthy and unhealthy, according to the VOR classification threshold value. The unhealthy and healthy class areas were 14.9 and 1.6 km^2^, accounting for 90% and 10% of the total area, respectively. The distributions of PS, FVC, and VOR were similar, even though FVC values were higher overall.

## 4 Discussion

### 4.1 Effect of grazing on vegetation

Grazing reduced the FVC and productivity, especially of palatable grasses and sedge species, and the AGB decreases with an increasing GI (Wu et al., 2009; Dai et al., 2019), consistent with our results (**Figure 2**, **Figure 3**). We also found no significant changes in species community diversity; instead, we observed a decrease in the PS, similar to the results obtained by Shi et al. in the northeastern TP (Akiyama & Kawamura, 2007; Shi et al., 2013). This is because grazing accelerates the loss of stem and leaf mass and nutrient cycling in these dominant herbivorous species (Semmartin et al., 2008). Selective plant feeding by livestock reduced the PS in the community, thereby altering the composition of the vegetation community, reducing the competitive ability of dominant palatable species, and allowing invasion by weedy or toxic grass to replace palatable species (Li et al., 2020), which is inconsistent with the results of Zheng et al., possibly because this study was conducted over a two-year period and did not set a larger grazing gradient, in which a moderate disturbance could increase the community diversity, while overgrazing led to a community diversity reduction (Zheng et al., 2012). Therefore, vegetation response to grazing disturbance is not only a volume reduction but also a vegetation composition change.

### 4.2 Driving factors of regional differences in VOR

Multiple regression analysis results showed that a GI increase caused a PS to decrease within the community, negatively affecting the VOR. Grazing allowed for the invasion of weeds or noxious weeds and, consequently, the deterioration of grassland health. Moreover, climatic factors also influenced the health of regional vegetation. The alpine meadow plant community has a relatively simple composition, being more susceptible to environmental changes (Gou et al., 2010; Sun et al., 2013; Zhang et al., 2015; Ran et al., 2019). The results showed that precipitation was a primary limiting factor for alpine vegetation dynamic rather than temperature. Suitable and sufficient precipitation positively affectd the health of regional vegetation (**Figure 6**). Our result was in line with the report of Fu et al. (Fu et al., 2018), suggesting that increased early summer precipitation had stronger effects on vegetation than did experimental warming in this alpine meadow site of the TP. Furthermore, precipitation is reportedly a critical factor controlling the primary productivity in most alpine grasslands (Shen et al., 2014; Sun et al., 2016).

### 4.3 Estimation of vegetation health

Specifically, we aimed to base our study on a vegetation indices (extracted from remotely sensed imagery) that is most sensitive to VOR, so that the VOR of the whole rangeland can be estimated efficiently and accurately. The results showed that the RVI was more accurate than other indices, suggesting that the RVI possessed a high degree of sensitivity to variations in the health of alpine meadow vegetation on the TP (**Table 2**, **Figure 5**). Here, ground and remote sensing data for the same period were used, and the VI was implemented as a proxy for an equation that converts measured *in situ* biomass into satellite data (Munyati et al., 2020). We included the most widely used NDVI as a reference indicator for studying the grassland VOR through an index model built based on the VOR and 11 VIs. Moreover, the RVI, known for its sensitivity, is included. And we also selected SAVI, MSAVI and OSAVI, which attenuate the soil background noise in different ways. All selected indicators have been extensively studied and are well known; however, most importantly, they use the commonly used Red(R) and Near Infrared (NIR) bands. The final VI most suitable for use in VOR in the study area was RVI, which agrees with the findings of Towers et al., who found that the RVI was the most sensitive of all the VIs tested (Towers et al., 2019). Furthermore, their results showed that the NDVI was the least sensitive, if accurate, to changes in the leaf area index among the indicators studied. However, this is in contrast with the findings of many scholars (Motohka et al., 2010; Prananda et al., 2020).

Healthy green vegetation has a large difference in NIR and R reflections, and RVI is aimed to enhance this difference by performing a ratio operation between NIR and R, thus enhancing vegetation information and reducing non-vegetation information. The RVI is a sensitive indicator parameter for green plants and correlates well with LAI, dry leaf biomass (DM), and chlorophyll content, and therefore identifies the proportion of community foragers well. Its discriminative power is influenced by the soil context and has a weak discriminatory power when the vegetation is less lush (Zhang et al., 2012). Therefore, it is more suitable for assessing the vegetation health of grasslands with a high FVC and toxic weed infestation in alpine meadows on the TP.

## 5 Conclusions

Although the vegetation index can be used to accurately estimate vegetation cover, since vegetation cover does not reflect the composition of vegetation communities. Specifically, without considering composition, a grassland can be falsely classified as healthy due to the confounding effects of invasive toxic weeds. The proportion of edible forage in a rangeland community can largely reflect the vegetation response of a rangeland under grazing disturbance. Therefore, we selected a comprehensive vegetation evaluation index, VOR, for a comprehensive evaluation of rangelands. VOR can not only indicate the quantity of pasture, but also characterize the quality of pasture. NDVI exhibited a closer relationship with vegetation cover, but the ratio vegetation index (RVI) showed a higher accuracy with VOR than soil-adjusted vegetation index (SAVI), Infrared percentage vegetation index (IPVI) and the commonly used Normalized difference vegetation index (NDVI). The Linear model between RVI and VOR presented the highest adjusted R^2^ values (0.357).

Therefore, it is necessary to choose more sensitive vegetation index to monitor the qualitative and quantitative changes of grassland health under disturbance, which will help to target the scientific and effective grazing management of grassland based on the spatial layout and dynamic changes of grassland health, and to realize the homogenization and sustainability of grassland utilization.

## Data availability statement

The original contributions presented in the study are included in the article/supplementary materials, further inquiries can be directed to the corresponding author/s.

## Author contributions

YH: conceptualization, methodology, software, writing original draft preparation, and data curation. XG: conceptualization, methodology, supervision, and writing-review and editing. AT: methodology. YC: conceptualization, methodology, funding-acquisition, supervision, and writing-review and editing. FH: methodology and resource. All authors contributed to the article and approved the submitted version.

## Funding

This study was funded by the Research Foundation for Advanced Talents of Inner Mongolia University (21800-5205103), the Inner Mongolia Grassland Talent (12000-12102517), the National Key Research and Development Program of China (2016YFC0501904-02).

## Acknowledgments

We thank Qingqing Ma and Guoli Zhou for their help with sampling.

## Conflict of interest

The authors declare that the research was conducted in the absence of any commercial or financial relationships that could be construed as a potential conflict of interest.

## Publisher’s note

All claims expressed in this article are solely those of the authors and do not necessarily represent those of their affiliated organizations, or those of the publisher, the editors and the reviewers. Any product that may be evaluated in this article, or claim that may be made by its manufacturer, is not guaranteed or endorsed by the publisher.

## Abbreviations

AGB, Above-ground biomass; EVI, Enhanced vegetation index; FVC, Fractional vegetation cover; GI, Grazing intensity; HG, Heavy grazing; IPVI, Infrared percentage vegetation index; LG, Light grazing; MSAVI, Modified soil-adjusted vegetation index; NDVI, Normalized difference of vegetation index; Pi_PS_, Importance value of each palatable species; PS, Palatable species; RDVI, Renormalized difference vegetation index; RVI, Ratio vegetation index; TP, Tibetan Plateau; VOR, Vigor, organization, and resilience.
